# Complement and Coagulation Cascade Activation Regulates the Early Inflammatory Mechanism of Resistance of Suckling Lambs Against *Haemonchus contortus*

**DOI:** 10.3390/pathogens14050447

**Published:** 2025-05-01

**Authors:** José Gabriel G. Lins, Alessandro F. T. Amarante

**Affiliations:** 1 Department of Veterinary Clinics, School of Veterinary Medicine and Animal Science, São Paulo State University (UNESP), Botucatu 18618-681, Brazil; jose.lins@unesp.br; 2 Department of Biostatistics and Biodiversity, Institute of Biosciences, São Paulo State University (UNESP), Botucatu 18618-689, Brazil

**Keywords:** innate immunity, homeostasis, nematode infection, pro-inflammatory response, sheep resistance

## Abstract

*Haemonchus contortus* is a highly pathogenic blood-sucking nematode from the abomasum of small ruminants. To develop effective control strategies, it is essential to understand the initial mechanisms involved in host resistance to this parasite. In this study, we used computational tools to analyze the complement and coagulation pathways generated from RNA sequencing of abomasal tissue from resistant (Santa Ines) and susceptible (Ile de France) young sheep artificially infected with *H. contortus*. Thirty-two differentially expressed genes annotated to the ovine genome were associated with the complement and coagulation cascades, of which 29 of them were overexpressed in Santa Ines. Our data identified potential markers for resistance trait selection in sheep, such as C3 (complement C3), F3 (tissue factor), F5 (coagulation factor V), CFB (complement factor B), and CFI (complement factor I). Santa Ines may have a more robust coagulation system, being activated by extrinsic pathways associated with tissue damage. The complement may act as a mediator of the innate immunity, and its activation in Santa Ines is associated with the classical, the lectin, and the alternative pathway. Finally, resistant Santa Ines lambs had a polygenic overexpressed architecture controlling both complement and coagulation cascades, which probably contributed to the early-onset protection against *H. contortus*.

## 1. Introduction

*Haemonchus contortus* is a pathogenic nematode from the abomasum of small ruminants, globally known for its multidrug resistance and responsible for causing high economic losses in sheep farming [1]. Understanding the biological mechanisms involved in the immunity of sheep against *H. contortus* is essential for the development of strategies that prevent disease. Aiming to trace a strategy for breeding resistant animals, the study of gene expression emerges as a sustainable and promising alternative for early animal selection in replacement of drug treatment.

Resistant Santa Ines lambs are able to mount a strong innate immune response against *H. contortus* infection at an early age (up to 68 days old), before weaning [2]. In our previous study, in which we evaluated histopathological alterations in the abomasal mucosa, we found that *H. contortus* infection stimulated hyperplasia of inflammatory cells and other inflammatory changes, including a greater mucosal thickness in the fundus of the abomasa in resistant Santa Ines lambs [2]. Among the cells identified in the marked inflammatory infiltrate were eosinophils and mast cells [2]. In a subsequent study, we performed RNA sequencing of the abomasal mucosa (fundic region) from resistant and susceptible suckling lambs, aiming to identify differentially expressed genes (DEGs) involved in the regulation of effector cells and to map the cell types involved in the immune response to infection, particularly those related to innate mucosa-associated immunity [3]. We identified 61 DEGs putatively linked to 15 distinct cell types, including B cells, CD4⁺ and CD8⁺ T cells, mucous cells, tuft cells, eosinophils, mast cells, and endothelial cells. Our analysis corroborated previous findings and revealed that resistance to *H. contortus* was genetically regulated and associated with a polygenic architecture that promoted an early, strong, and effective cellular response [3]. However, in that study, KEGG pathway enrichment analyses were not performed to further investigate the functional pathways underlying these cell recruitment profiles.

By evaluating the dynamics of the immune functional mechanisms promoting resistance to *H. contortus* in suckling lambs, through Gene Ontology and Kyoto Encyclopedia of Genes and Genomes (KEGG) enrichment analyses, we found that, in resistant lambs, a more intense inflammatory response and an early and strong cellular response were accompanied by an effective tissue repair process [4]. This process, driven by fibroblast proliferation, collagen production, and epithelial maturation, contributes to the maintenance of the mucosal integrity in resistant lambs. Additionally, the complement and coagulation cascades ranked among the top 20 enriched KEGG pathways in suckling lambs infected with *H. contortus*, suggesting that their early activation may drive stronger and earlier inflammatory response in resistant lambs [4].

Complement and coagulation systems are intimately related and share important features that prevent the establishment of pathogens in the very early stage of an infection [5]. Additionally, it is supposed that these systems play key roles in sheep protection for *H. contortus* and are essential for the development of effective innate and adaptive immune responses against invading pathogens [6,7,8]. The complement system, involved in innate immunity, acts as one of the main mechanisms against pathogens, triggering the recruitment of effector cells to control the infection [2,9].

*Haemonchus contortus* alters the mucosal homeostasis during infection, inducing strong immune-modulatory responses in immunocompetent hosts [10]. Three different pathways are involved in the activation of the complement cascade, the classical, alternative, and lectin, which depend on the initial trigger or recognized target molecule. Despite their different activation mechanisms, all of them converge toward a common goal: the elimination of the pathogen [11]. During parasitic infection, the complement cascade may be mostly activated by the classical pathway, in which the recognition of the pathogen-associated molecular patterns (PAMPs) by specialized receptors, such as the toll-like receptors (TLRs), which promote pathogen uptake and initiate an intracellular cascade in host immune cells [4,10,12]. An important strategy for successful *H. contortus* establishment is the capacity for evading the immunological mechanisms of the host, including the host TLRs [13]. In this context, the lectin pathway is activated by the expression of important animal lectins, such as mannose-binding lectin and ficolins, which are involved in innate immunity [14].

Furthermore, to gain deeper biological insights into the molecular mechanisms related to the complement and coagulation cascades and their influence in regulating the immunological mechanism involved in the degree of resistance of Santa Ines hair sheep and Ile de France wool sheep against *H. contortus,* we integrated KEGG pathway and protein–protein interaction (PPI) analyses in this study. KEGG analysis is applied to identify biological pathways enriched among DEGs, while PPI analysis, based on the STRING database, is applied to reveal key interaction networks and hub genes involved in these processes. The STRING database (https://string-db.org/) integrates protein–protein interaction analyses, in both physical and functional associations [15]. This combined approach may provide information regarding potential targets for developing genetic or therapeutic strategies to enhance parasite resistance, even in susceptible breeds.

The results reported here will contribute to a better understanding of the very-early-onset immunity of lambs against *H. contortus* infection and guide us in future molecular studies, with the aim of developing diagnostic biomarkers useful for breeding selection for resistance.

## 2. Materials and Methods

Details about the experimental design and the artificial *H. contortus* infections of the animals involved in this study are described by Lins et al. [2]. In summary, purebred Santa Ines and Ile de France ewes in the first third of pregnancy were acquired from commercial flocks in the state of São Paulo. In the experimental area, naïve lambs were born, housed individually with their ewes after lambing, and were kept worm-free until the start of experimental infections, when they reached 14 days of age (day 0 of the first infection). Naïve Santa Ines (*n* = 4) and Ile de France (*n* = 4) suckling lambs were orally infected with infective larvae (L3) of *H. contortus* every two days until the age of 66 days, following an infection protocol consisting of 27 infections, as follows: (1) nine infections with 100 L3 each, (2) nine infections with 200 L3 each, and finally, (3) nine infections with 300 L3 each (total of 5400 L3). Two days after the last infection (at 68 days of age), the lambs were euthanized, their abomasa were removed, and tissue samples from the fundic region of four Santa Ines and four Ile de France suckling lambs were collected for mRNA extraction and sequencing [3].

Briefly, total RNA extraction from abomasa fundus tissue was conducted using the RNeasy^®^ Mini Kit (Qiagen, Valencia, CA, USA), following the manufacturer’s instructions. RNA concentration and integrity were determined using a Quantus™ Fluorometer (Promega, Madison, WI, USA) and an Agilent RNA 6000 Nano Kit^®^ with an Agilent Technologies 2100 Bioanalyzer (Agilent, Palo Alto, CA, USA), respectively. For cDNA preparation before sequencing, Poly (A) tail-containing mRNAs were purified using oligo-(dT) magnetic beads and fragmented into small pieces using a divalent cation buffer at elevated temperature. Additionally, Poly (A) RNA libraries were prepared following Illumina’s TruSeq-stranded-mRNA sample preparation protocol (Illumina, San Diego, CA, USA). The quality and quantity of the sequencing library were assessed using the High Sensitivity DNA Chip on an Agilent 2100 Bioanalyzer (Agilent Technologies, Palo Alto, CA, USA). Subsequently, paired-end sequencing with 150 bp read length was carried out on an Illumina NovaSeq 6000 platform (Illumina, San Diego, CA, USA) [3].

Prior to transcript sequence analysis, raw paired-end sequencing reads were processed to obtain valid (high-quality) reads using Cutadapt (version 1.10) [16] and custom in-house Perl scripts [3]. FastQC (version 0.10.1) was used for assessing sequency quality, and then the filtered valid reads were aligned to the *Ovis aries* v4.0 reference genome (accession ID: GCF_000298735.2; https://www.ncbi.nlm.nih.gov/assembly/GCF_000298735.2/ (accessed on 6 January 2020) using HISAT (version 2.0) [17]. Mapped reads of each sample were assembled using StringTie (version 1.3) [18]. After a comprehensive transcriptome was generated, the transcript estimation levels were quantified using StringTie (version 1.3) [18] and the edgeR R Bioconductor package (version 3.18.1/date 29 September 2016) [19]. Finally, DEGs between infected Santa Ines and Ile de France lambs were identified using StringTie (version 1.3) [18], based on fragments per kilobase of exon per million mapped (FPKM) reads. All these bioinformatic tools and analytical procedures are described in detail by Lins et al. [3].

Genes were considered expressed when they were observed at at least 0.01 FPKM. DEGs between breeds were initially selected based on *p* < 0.05, followed by a corresponding adjusted *p* value, in which they were subjected to false discovery rate (FDR) correction using the Benjamini and Hochberg method (edgeR), considering those with an FDR threshold (adjusted *p*-value) < 0.05 [3].

### 2.1. Data Set Recovery, Data Analysis, and Bioinformatics

A total of 1146 DEGs were identified between Santa Ines and Ile de France lambs based on RNA-seq data, using a statistical significance threshold of *p* < 0.05. For KEGG pathway enrichment analyses, only DEGs that met the criteria of |Normalized Enrichment Score (NES)| > 1, *p*-value < 0.05, and false discovery rate (FDR) *q*-value < 0.25 were considered significantly enriched. After applying these filters, 781 DEGs with FDR < 0.25 were selected for GO and KEGG enrichment analyses [20,21,22], using the GO database (http://www.geneontology.org, accessed on 15 March 2025) [23,24] and the KEGG database (https://www.kegg.jp/, accessed on 15 March 2025) [25]. All analytical procedures are described in detail by Lins et al. [4].

For this study, we explored the KEGG (Kyoto Encyclopedia of Genes and Genomes) enrichment analysis dataset of differentially expressed genes (DEGs) between Santa Ines and Ile de France lambs annotated to the *Ovis aries* v4.0 reference genome, and we selected the complement and coagulation cascades pathway (oas04610) for conducting a detailed computational analysis. Furthermore, we chose this pathway because it was among the 20 most significantly enriched KEGG pathways found by Lins et al. [4], driving us to hypothesize that the early activation of these cascades may regulate the more intense and earlier inflammatory response observed in resistant lambs. Thus, identifying the DEGs involved in these pathways and understanding their regulation could provide promising targets for monitoring the resistance trait in the very initial stage of the infection.

The ClustVis tool (https://biit.cs.ut.ee/clustvis/, accessed on 15 March 2025) was used for visualizing patterns and relationships between samples and between DEGs annotated to the complement and coagulation cascades pathway (oas04610) [26].

The selection of “top hit” genes and their ranking were performed according to the Euclidean distance [27]. The analysis of top hit genes identifies the DEGs that present the highest expression variation between breeds (Santa Ines vs. Ile de France). In addition, this analysis can generate new hypotheses about the role of these genes in relevant biological processes.

### 2.2. Protein–Protein Interaction (PPI) Network

The analyses of protein–protein interactions (PPI) were performed to explore the functional (indirect) associations at the protein level, between the 32 DEGs related to the complement and coagulation cascades pathway. The PPI network analyses were performed using Cytoscape [28] version 3.8.2 with the plugins STRING version 1.7.0 (Search Tool for the Retrieval of Interacting Genes/Proteins database version 11.5, http://string-db.org, accessed on 15 March 2025), NetworkAnalyser [29] version 4.4.8, MCODE (Molecular Complex Detection) [30] version 2.0.0, and CytoHubba [31] version 0.1.

STRING was used for recovering predicted protein–protein interactions for *Ovis aries*. A threshold of >0.04 was used as a confidence score for functional association among proteins. Data from String were associated with the organism *Ovis aries* (NCBI taxonomy: Id.:9940) [32]. The MCODE plugin was used to find densely connected (highly interconnected) regions in each network obtained from the selected DEGs. An MCODE score > 2.5 was set as the cuttoff criteria, with the default parameters (Degree cuttoff ≥ 2, Node score cuttoff ≥ 0.200, K-score ≥ 2, and Max depth = 100). The NetworkAnalyser plugin was used for calculating topological properties (such as degree distribution, clustering coefficient, and centrality) of the main network obtained from STRING. Furthermore, the CytoHubba plugin was utilized to predict and explore the 10 most common PPI network hub genes according to the Maximal Clique Centrality (MCC) [33].

## 3. Results

At the end of the experimental period (68 days of age), Santa Ines lambs had a mean eggs per gram of feces (EPG) rate of 1200, ranging from 0 to 3600 (with two lambs having 0 EPG—Resistant 2 and Resistant 4), while Ile de France lambs had a mean of 11,075 EPG, ranging from 5600 to 15,500 EPG.

A total of 1146 DEGs between Santa Ines and Ile de France lambs were identified from the RNAseq dataset with statistical significance at *p* < 0.05 [3]. From those, 781 genes with FDR < 0.25 were subjected to KEGG enrichment analysis [4], and a total of 32 differentially expressed genes between breeds (Santa Ines vs. Ile de France) were related to the complement and coagulation cascades pathway (oas04610, *p* value = 1.36 × 10^−8^), in which 29 were overexpressed in the resistant Santa Ines breed (Supplementary File S1).

**Figure 1:**
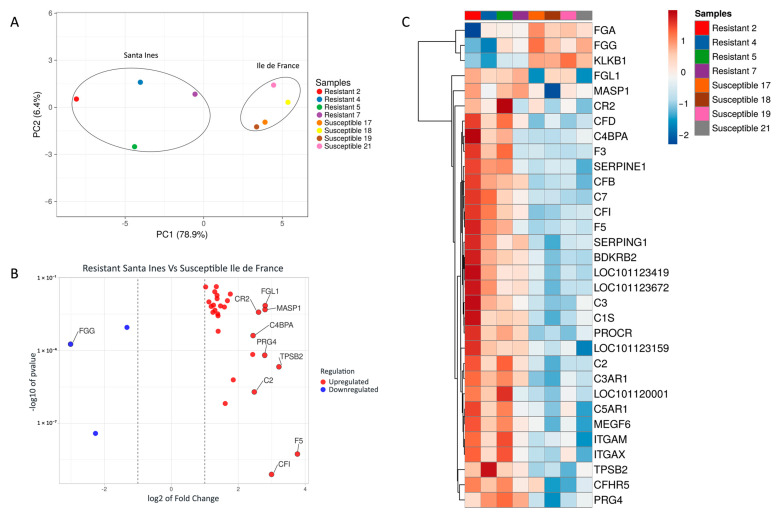
(**A**) Principal component analysis (PCA) of differentially expressed genes (DEGs) between resistant Santa Ines (SI) and susceptible Ile de France (IF). Ellipses were manually added. (**B**) Scatter plot of the 32 DEGs in abomasal samples. Blue points in scatterplot represent the downregulated genes, and red points represent upregulated genes. Regulation is related to Santa Ines lambs. (**C**) Heatmap of differentially expressed genes (DEGs) of Santa Ines versus Ile de France lambs experimentally infected with *Haemonchus contortus*. All genes reported are related to the coagulation and complement cascades pathway. Each row represents a gene, and each column a sample (among the Santa Ines breed, lambs 2 and 4 were the most resistant).

The resistant and susceptible sheep breeds were grouped into two separate clusters, as expected. A higher variability was observed among the Santa Ines lambs compared to the Ile de France lambs. The PCA of the gene set demonstrated a clear separation between the Santa Ines and Ile de France lambs, explaining 78.9% and 6.4% of the variance of gene expression between breeds, respectively (see Figure 1A).

The top hit genes were calculated based on the Euclidean distance among the 32 differentially expressed genes in abomasal samples, and the top 10 hit genes are reported in Figure 1B. The heatmap showed that resistant Santa Ines had higher variation in the pattern of gene expression among the individuals compared to susceptible Ile de France lambs (see Figure 1C).

**Figure 2:**
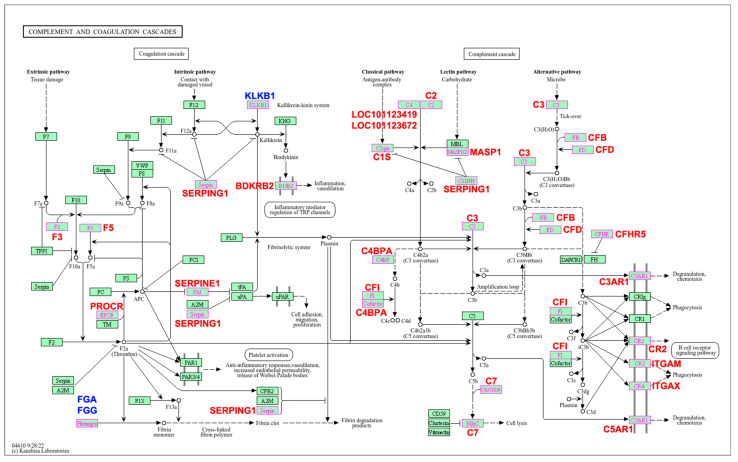
Biological pathway map of the complement and coagulation cascades significantly enriched in response to *Haemonchus contortus* infection in experimentally infected Santa Ines and Ile de France suckling lambs. Highlighted genes represent those differentially expressed between breeds. Additionally, those in red are upregulated and those in blue are downregulated. Regulation is related to resistant Santa Ines lambs. Adapted for academic purposes with Copyright Permission (number 251138) from Kanehisa Laboratories [25].

Twenty-six DEGs were highlighted in the pathway map viewer developed by Kanehisa Laboratories for the complement and coagulation cascades pathway (Figure 2), while the remaining six DEGs were not included in the map or were classified as ‘gaps’ (Supplementary File S1). Typically, ‘gaps’ represent non-essential genes or genes whose role in the pathway is not yet fully understood.

**Figure 3:**
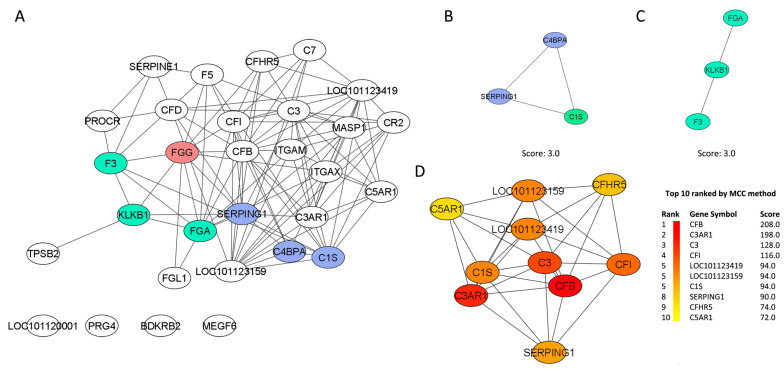
(**A**) The network demonstrates the protein–protein interaction (PPI) drawn from the 32 genes found differentially expressed in the abomasal tissue of Resistant Santa Ines and Susceptible Ile de France suckling lambs experimentally infected with *Haemonchus contortus*. The network belongs to the enriched “complement and coagulation cascades” pathway. The nodes are represented as ellipses (white, green, and blue), and the edges (black) as lines. The red gene represents the one with the highest number of betweenness centrality in the PPI network. Cluster 1 (**B**) and cluster 2 (**C**) are derived from the network of interactions between protein and protein. Clusters were selected using the molecular complex detection (MCODE) plugin. (**D**) The first 10 genes (hub genes) of the MMC method were chosen using the CytoHubba plugin and are represented as elliptical nodes, and the more forward ranking is represented by a redder color.

The PPI network is presented as the full STRING network (the edges indicate both functional and physical protein associations). The PPI enrichment *p*-value was <1.0 × 10^−16^, indicating that these proteins are not random. The meaning of network edges was based on the confidence and strength of data support. The summary statistics of the PPI network showed that the 32 imputed DEGs generated a list of 30 nodes, 106 edges, and a clustering coefficient of 0.487 (Figure 3A). The FGG gene had the highest number of betweenness centrality (0.0924), indicating that this node had the highest number of bridges along the shortest paths.

MCODE plugging selected two clusters, and both had a score of 3.0. Cluster 1 (Figure 3B) was composed of three nodes (C4BPA, SERPING1 and C1S) and three edges. Cluster 2 (Figure 3C) was also composed of three nodes (KLKB1, FGA and F3) but two edges. The clustering coefficients of both clusters were not calculated by NetworkAnalyser because of the number of nodes (<4). The gene C1S was identified as the seed (MCODE score 3.444) of the cluster, while in the second cluster, the seed gene was not predicted because the genes had a similar MCODE score. The MMC method ranked the top 10 protein-related genes, taking into consideration the closeness centrality number (Figure 3D).

## 4. Discussion

Coagulation and complement cascades are closely related and dependent, and these cascades seem to play an important role in the host defense against *H. contortus* infection. Our data identified several genes that differ in their expression level between breeds and that may be used as possible markers of the complement and coagulation pathway, possibly also in other commercial sheep breeds. The resistant line of Merino sheep under *H. contortus* infection had 24 genes associated with complement and coagulation cascades, which were significantly upregulated in comparison to the susceptible line flock [8].

The complement and coagulation systems act through tissue injuries and inflammation [34]. Blood coagulation may occur in response to the activation of two pathways, the extrinsic and intrinsic [35]. The extrinsic is activated by tissue damage, while the intrinsic is activated by the contact with damaged vessels. *H. contortus* is known for causing high tissue damage in the abomasal mucosa, mainly in the histotrophic phase, during the development into the L4 stage [36]. Infected lambs had greater alteration in the abomasal mucosa in comparison with non-infected control lambs. However, when comparing the two infected breeds, Ile de France lambs had more irregularities in the mucosa of fundus, with the presence of microhemorrhages through the mucosa [2]. In addition, red petechiae were macroscopically observed on the surface of the abomasal mucosae, and microscopically, there was the presence of erythrocytes free in the gland crypts and also on the surface of the mucosa, mainly in infected Ile de France lambs. Such findings are in accordance with those reported by Charleston [37] in the mucosae of sheep experimentally infected by *H. contortus*.

Upregulation of the genes F3 and F5 in Santa Ines lambs appeared to enhance the activation of the coagulation cascade in response to tissue damage. During the infection inside the host, immature larvae, mainly the fourth stage larvae, up to the final molt, may cause glandular congestion and hemorrhage into the abomasum [38]. The coagulation cascade is a very important pathway, mainly during the inflammatory responses caused by pathological processes. Thrombin converts soluble fibrinogen to fibrin clots and contributes to the activation of protease-activated receptors, involved in innate response [39,40]. Hemostasis is involved in the process of blood clotting formation and fibrinolysis [41,42]. Santa Ines lambs had a higher expression of genes related to hemostasis [9]. Coagulation factor V (F5) was found upregulated for Santa Ines lambs, suggesting its key importance in fibrin clot formation [43].

Fibrinogen is important for blood clot formation. Ile de France lambs had higher expression of the three DEGs involved in the production of fibrinogen alpha and gamma chains (FGA and FGG) in comparison with Santa Ines lambs. This process may be important for susceptible sheep breeds, since these encoded preproproteins are proteolytically processed by thrombin during the conversion of fibrinogen to fibrin. FGG emerged as the most central gene in the PPI network, even though it was downregulated in the Santa Ines breed; however, this is not problematic. As shown in Figure 1C, two Santa Ines lambs (Resistant 2 and Resistant 4) exhibited the most pronounced FGG downregulation within the group. Coincidentally, these lambs had zero eggs per gram of feces at 68 days of age and were harboring very low *Haemonchus* burdens (20 and 42 parasites, respectively). Given their low worm counts, why would these animals require increased expression (upregulation) of FGG? The lower the number of parasites, the lower the number of lesions caused in the mucosa, thus resulting in reduced hemorrhage during blood feeding and a reduced need for clotting activity. Supporting this, the other two resistant Santa Ines lambs (Resistant 5 and 7) with higher parasite burdens (496 and 1003 parasites, respectively) showed higher expression levels of FGG. Fibrinogen not only plays a central role in coagulation but is also increasingly recognized as a key modulator of immune and inflammatory responses [44]. Another hypothesis is that the observed downregulation of FGG reflects condition-dependent suppression, potentially indicating a controlled immune profile. This may represent a compensatory or regulatory mechanism in response to altered inflammatory signaling to avoid or minimize host tissue damage and contributing to tolerance against the parasite.

Furthermore, our results showed that Ile de France had a decreased complement activation, which probably contributed to a delayed inflammatory response initiation. During *H. contortus* infection, effector molecules from the complement system will result in the recruitment of inflammatory and immunocompetent cells to the site of host–parasite interaction, with the aim of limiting the infection [45]. The increased number of platelets, endothelial cells, and mast cells may influence the regulation of coagulation [35,46]. Resistant Santa Ines lambs showed a higher number of mast cells in abomasal mucosa and submucosa than susceptible Ile de France lambs and also showed higher expression of DEGs related to mast cells and endothelial cells [2,3], with a robust tissue repair mechanism and a strong smooth muscle contraction [4]. All these previous findings give us supportive evidence that Santa Ines may present a more robust coagulation system and that this cascade contributes to the early-onset protection against *H. contortus*. C5A may be related to the chemotactic activity of neutrophils. The gene C5AR1 was differentially expressed between breeds and was upregulated for resistant Santa Ines. This gene is related to cellular degranulation and chemotaxis. Lins et al. [3] reported that the recruitment of neutrophils to the abomasal mucosa was influenced by the overexpression of several genes that were differently expressed in comparison to susceptible Ile de France. CFB is a single polypeptide associated with the alternative complement pathway and has been associated with the sheep Major Histocompatibility Complex (MHC) [47]. The expression of the genes C7 and CFI were affected by *H. contortus* infection, and in accordance with our results, they were overexpressed for resistant Canaria hair sheep in comparison with susceptible sheep [48].

According to Oikonomopoulou et al. [35] the complement and coagulation cascades may act as an important defense against pathogens, and any dysregulation in the cascades may result in the clinical manifestation of diseases such as sepsis. Keawy et al. [9] reported the important role of the complement and coagulation cascades in the tissue repair system and maintenance of homeostasis. It is possible that molecules produced by the parasite may interfere with these protective systems in Ile de France lambs, which does not occur in Santa Ines lambs. As a parasite strategy, for example, *H. contortus* produces a serpin with anticoagulant properties, which may contribute to its feeding mechanism. In vitro studies have shown that this serpin can inhibit host coagulation, although whether this occurs through competitive inhibition or another mechanism remains unclear [49,50].

The complement system may act as a mediator of the innate immune response, and its activation is associated with three pathways: the classical, the lectin, and the alternative pathway.

A secreted protein by *H. contortus* calreticulin (CalR) has been reported to inhibit the activation of the complement through the classical pathway, probably due to binding attachment to the Complement component 1q (C1q) protein [51]. In this study, Santa Ines lambs showed upregulation of the C1S gene, and PPI analyses show this gene as an important node in the network, suggesting that the C1 complex (C1qrs) may mitigate the immune modulation induced by *H. contortus* through activation of the classical complement pathway, leading to pathogen opsonization and activation of host immune cells.

The lectin pathway seems be involved in the activation of the complement cascade in resistant Santa Ines through the overexpression of the genes MAPS1, SERPING1, C2 and LOC101123419. The lectin pathway is important in the transition between the complement and coagulation systems. Additionally, MASP1 (mannan-binding lectin serine) upregulation indicates that this lectin may act in concert with TLRs, through the recognition of PAMPs exposed on pathogens [14,52]. TLR2 was overexpressed for resistant Santa Ines compared to Ile de France lambs and was associated with putative macrophages and neutrophils in abomasal mucosa [3]. Such results suggest that Santa Ines lambs are able to overcome host immune-modulation by *H. contortus* excretory-secretory product through the activation of all the three complement pathways. Proteomic analyses showed that *H. contortus* excretory-secretory proteins interact with host T cells, impairing their viability, inhibiting their proliferation, and interfering with normal cell cycle development [53].

Additionally, MASP1 may be involved in the clavering of C2 and C4, which enables the formation of C3 convertase, a converging point among the three complement activation pathways [43,54]. The protein glyceraldehyde-3-phosphate dehydrogenase (GAPDH), derived from *H. contortus* excretory/secretory products, acts by negatively regulating the complement cascade in the host, avoiding its activation through the bond to the C3 protein [55]. Santa Ines lambs showed higher expression of the C3 gene, driving us to believe that in Ile de France lambs, the C3 protein was not sufficiently produced/expressed to overcome the *Haemonchus* GAPDH binding mechanism, which compromised the activation of the complement through the alternative pathway.

The alternative pathway is as important as the other two pathways for the complement, and it is triggered by the spontaneous activation of the central recognition molecule C3 [5,35]. The alternative pathway promotes the depletion of the C3 component as a mechanism that promotes a quick amplification of the pro-inflammatory responses; C3 products are reported to be involved in the opsonization of the parasites during infection [11]. Additionally, the alternative pathway is crucial for the amplification of the complement, regardless of the method of initiation of the cascade [10].

Santa Ines lambs were more capable of maintaining an effective complement activation even in the presence of *H. contortus*-derived inhibitors. In contrast, Ile de France sheep show a more pronounced suppression of complement-related genes/proteins during infection, indicating that *H. contortus* immune evasion is more effective in this breed. This may partially explain their higher susceptibility and parasite burden under infection.

## 5. Conclusions

Resistant Santa Ines lambs had a polygenic architecture controlling both complement and coagulation cascades through the activation of the following networks—the lectin pathway, the classical pathway, and the alternative pathway of complement activation—as well as through the extrinsic pathways associated with tissue damage.

We provide evidence that the complement and coagulation cascade activation is strongly affected by *H. contortus* infection and that resistant lambs may have highly overexpressed genes regulating this pathway. Further studies are necessary for validating the markers for the resistance trait in other sheep populations and also for investigating the potential therapeutic target for early modulation of an effective immune onset within the complement and coagulation cascades, as it was associated with initiating the inflammatory response against the infection caused by *H. contortus*.

## Data Availability

The datasets supporting the conclusions of this article are included within the article. RNAseq data used in this study were recovered from NCBI Sequence Read Archive (BioProject accession number PRJNA851745).

## Supplementary Materials

The following supporting information can be downloaded at: https://www.mdpi.com/article/10.3390/pathogens14050447/s1, Supplementary File S1: Gene expression differences in lambs infected with *Haemonchus contortus*, focusing on complement and coagulation cascades.
